# Magnetocaloric effect modeling of dysprosium-transition metal based intermetallic alloys for magnetic refrigeration application using hybrid genetic algorithm based support vector regression intelligent method

**DOI:** 10.1371/journal.pone.0298431

**Published:** 2024-02-06

**Authors:** Sami M. Ibn Shamsah

**Affiliations:** Department of Mechanical Engineering, College of Engineering, University of Hafr Al Batin, Hafr Al Batin, Saudi Arabia; Chandigarh University, INDIA

## Abstract

Intermetallic alloy containing rare earth dysprosium ions with the associated unfilled 4f shell electrons and sub-lattice of 3d-transition metal, results into fascinating magnetic properties which are useful for green refrigeration technological application. Magnetocaloric effect remains the fundamental principle upon which magnetic refrigeration technology is based while this cooling technology has advantages of cost effectiveness, high efficiency and environmental friendliness as compared with the existing conventional gas compression systems. Maximum magnetic entropy change (which controls the hugeness of magnetocaloric effect) of intermetallic alloy Dy-T-X (where T = transition metal and X = any other metal or nonmetal) is modeled in this work using hybrid genetic algorithm based support vector regression (GSVR) computational intelligent method with applied magnetic field, ionic concentration and ionic radii descriptors. The developed GSVR-G model with kernel Gaussian function outperforms GSVR-P model with polynomial function with improvement of 85.23%, 78.82% and 78.67% on the basis of the computed correlation coefficient (CC), mean absolute error (MAE) and root mean square error (RMSE) on testing sample, respectively. The developed model further investigates the influence of applied external magnetic field on magnetocaloric effect of DyCuAl intermetallic alloy. The developed models in this work circumvent experimental challenges of magnetocaloric effect determination while the recorded precision of the developed model further opens doors for possible exploration of these intermetallic compounds for addressing environmental challenges associated with the present system of cooling.

## 1.0. Introduction

Immense potentials of magnetic materials in solid state green refrigeration have lately attracted significant attention in large magnetocaloric effect based materials [1, 2]. Advantages of this magnetic refrigeration system are enormous as compared to the existing conventional gas compression systems with characteristic lower efficiency and global warming treat due to utilization of environmental toxic chemicals [3–5]. Additionally, utilization of solid refrigerants contributes to the compaction of magnetic refrigeration as compared to the mentioned conventional system of refrigeration [6]. Structural magnetic rearrangement and alteration in magnetic state occur when magnetic field is externally and adiabatically applied to or removed from magnetic materials. This leads to change in temperature of the magnetic materials and the process of the temperature or entropy change due to applied field is referred to as magnetocaloric effect [7]. Recent research areas focused on developing magnetic materials with huge magnetocaloric effect for this technology [8–12]. Although, virtually all magnetic materials exhibit magnetocaloric effect while the magnetism of the magnetic materials and magnetic transition phase drive the strength of the exhibited magnetocaloric effect. Transition metals are characterized with weak magnetism or total absence of magnetism. The magnetism of transition metals leads to higher ordering temperature with reduced magnetic moment. 4f shell electrons which are partially unfilled are the backbones of the observed magnetism in rare earth metals such as dysprosium (Dy) [13]. Rare earth intermetallic compounds exhibit large magnetocaloric effect over a wide range of temperature [14, 15]. The hugeness of magnetic moment becomes more pronounced with reduced ordering temperature for well localized electrons. Other significant features of these compounds that promote potential cryogenic refrigeration applications include good resistance to corrosion, high electrical conductivity and low magnetic hysteresis. Therefore, alloys combining rare earth metals with transition metals become fascinating in terms of magnetism especially when the desired application is based on hugeness of magnetocaloric effect.

Electronic structure of dysprosium -transition metal based intermetallic compounds involves the combination of sub-lattice 4f dysprosium ions with unfilled 4f shell and 3d sub-lattice of transition metals [16]. These features translate to high magneto-crystalline anisotropy and excellent magnetic moments [17]. Rare earth based intermetallic compounds usually have huge magnetocaloric effect over a wide magnetic temperature range as compared with other crystalline materials that demonstrate large magnetocaloric response over a narrow range of temperature [18]. Many intermetallic compounds of rare earth -transition metals are known to have magnetic moments which are ordered ferromagnetically within a region of low temperature and demonstrate magnetic phase transition of second-order nature [19]. The exchange interaction between dysprosium and some transition metals such as nickel might be weak and results into remarkable magnetocaloric effect upon combination. These kind of intermetallic compounds have potential interesting application in cooling technology [20]. Many research outputs have shown that combining rare earth metals in alloys with other metals and non-metals can significantly enhance magnetocaloric response of the alloy and strengthen possible implementation in this green technology [21]. This work models the maximum magnetic entropy change (which controls the refrigerant’s magnetocaloric effect) of dysprosium based intermetallic alloy (Dy-T-X where Dy = dysprosium, T = transition metal and X = any other metal or nonmetal) using hybrid genetic algorithm based support vector regression computational method with ionic radii molecular descriptors.

Support vector regression (SVR) is a novel technique of pattern acquisition through leaning and governed by operational theory of statistical learning [22]. The algorithm conveniently handles systems with characteristic non-linearity through structural risk principle of minimization where generalized error is minimized through convex optimization solving after Lagrange multipliers implementation [23–25]. The algorithm extends to wide application domain as a result of its novelties, simplicity and precision [26–28]. The choice SVR algorithm (for modeling the magnetocaloric effect of dysprosium-transition metal based intermetallic alloys) among all other available intelligent algorithms is due to its unique features in establishing or approximating relations between the descriptors and the target with reasonable degree of accuracy using relatively few data-samples. Regression precision and accuracy of the algorithm is significantly influenced by some tunable parameters within SVR algorithm [29]. Genetic algorithm is hybridized with SVR algorithm for the purpose of ensuring optimal combinatory selection of these parameters. Genetic algorithm is an evolutionary algorithm that addresses optimization problems that have characteristic wide range of uncertainties [30, 31]. The choice of genetic algorithm is due to its simplicity and effectiveness in searching complex solution spaces. The algorithm uses data structure of chromosome-like manner to code biological evolution processes. Genetic algorithm employs selection, crossover and mutation operations to replace existing population with new offsprings of better qualities. The model parameters optimized by genetic algorithm in this work include the penalty factor, epsilon and kernel parameter of the chosen mapping function.

The developed genetic algorithm based support vector regression model (GSVR) with Gaussian mapping function (GSVR-G) demonstrates enhanced performance as compared with GSVR-P that employs kernel polynomial using parameters that evaluate and assess model performance. The performance assessment parameters implemented include correlation coefficient (CC), mean absolute error (MAE) and root mean square error (RMSE). For training dysprosium -transition metal based compounds; GSVR-G performs better than GSVR-P with improvement of 30.76%, 68.49% and 77.25% for CC, MAE and RMSE parameters, respectively while respective improvements of 86.23%, 78.82% and 78.67% were obtained on testing data samples. Effect of applied field on DyCuAl alloy was established using developed model.

The remaining sections of the manuscript are organized as follow: section two formulates the mathematical backgrounds of support vector regression algorithm and that of genetic algorithm. Section three details the computational methodology with data sample descriptions. Section four discusses the results of the modeling and simulation. The convergence of genetic algorithm is presented while the two developed models are compared and also presented. Results of the effect of external field variation on maximum magnetic entropy change are also presented. Section five summarizes the entire manuscript.

## 2.0. Algorithm formulations and background

The formulations of both intelligent and optimization algorithms are presented in this section. Mathematical details of support vector regression and biological navigation principles of genetic algorithm are presented.

### 2.1. Support vector regression description

Support vector regression (SVR) successfully handles and models non-linear systems due to the strong fundamental structural risk minimization principle adopted by the algorithm [32, 33]. Consider magnetocaloric compounds of dysprosium -transition metal based data samples $d={\left(\sigma_{k},\eta_{k}^{*}\right)}_{k}^{m}$ in which *σ*_*k*_ are the input descriptor vectors while *η* is the associated measured magnetocaloric effect. The algorithm considers the estimated function presented in Eq (1) for attainment of its objectives [34].


η(σ)=〈ω,σ〉+θ (1)

Where *η* is the predicted maximum magnetic entropy change using SVR algorithm. The unknown coefficients *ω* and *θ* which respectively stands for the weight vector and bias are estimated through minimization of the risk function shown in Eq (2).


$$r(\eta )=\frac{\mu }{m}\sum_{k=1}^{n}I(\eta (\sigma_{k})-\eta_{k}^{*})+\frac{{\left\|\beta \right\|}^{2}}{2}$$ (2)

Where ‖*β*‖^2^ represents the Euclidean norm, *I* is the *ε*−*insensitive* loss function shown in Eq (3) while *μ* is the regularization factor. The regularization factor assigns penalty for the sample with deviation exceeding the error epsilon *ε* threshold. This factor is a constant and non-zero value defined by the user and can be tuned using manual or evolutionary algorithms.


$$I\left(\eta (\sigma )-\eta^{*}\right)=\{\begin{array}{cc} \|\eta (\sigma )-\eta^{*}\|-\varepsilon & |\eta (\sigma )-\eta^{*}|\geq \varepsilon \\ 0 & \eta (\sigma )-\eta^{*}<\varepsilon \end{array}$$ (3)

Inclusion of slack variables (*ζ* and ζ*) become necessary especially during potential risk of exceeding error threshold. The slack variables dictate the distance between the measured values and *ε*-tube boundary values. Slack variables are employed when the achievement of constraints becomes difficult while error epsilon threshold is among the tunable parameters in SVR algorithm and controls the generalized error bond of the predictive model. The resulted dual problem is solved using optimization approach shown in Eq (4) [35, 36].


$$Minimize:\frac{1}{2}\sum_{k}^{m}\sum_{i}^{m}(\chi_{k}-\chi_{k}^{*})(\chi_{i}-\chi_{i}^{*})\gamma (\sigma_{k},\sigma_{i})+\sum_{k=1}^{m}\eta_{k}^{*}(\chi_{k}-\chi_{k}^{*})-\varepsilon \sum_{k=1}^{m}\left(\chi_{k}+\chi_{k}^{*}\right)$$ (4)

The minimization problem shown in Eq (4) is subjected to the constraints depicted by Eq (5).


$$\sum_{k=1}^{m}\left(\chi_{k}+\chi_{k}^{*}\right)=0,\chi_{k},\chi_{k}^{*}\in [0,\mu ]$$ (5)

Where *χ*_*k*_ and $\chi_{k}^{*}$ are Lagrange multipliers employed for optimization problem solution. The input vectors associated with non-zero coefficients of the optimization solutions are referred to as support vectors. With the implementation of the aforementioned optimization function, the expression representing the predictions of SVR algorithm is presented in Eq (6).


$$\eta (\sigma )=\sum_{k=1}^{m}\left(\chi_{k}-\chi_{k}^{*}\right)\langle \sigma_{k},\sigma \rangle +\theta$$ (6)

The kernel function maps the original problem to linear problem in high dimensional space. This mapping function is represented in Eq (7) while the final non-linear estimation regression equation of SVR algorithm is presented in Eq (8)

$$\gamma (\sigma_{k},\sigma_{i})=\phi (\sigma_{k})\phi (\sigma_{i})$$ (7)

$$\eta (\sigma )=\sum_{k=1}^{m}\left(\chi_{k}-\chi_{k}^{*}\right)\gamma \left(\sigma_{k},\sigma \right)+\theta$$ (8)

In this problem, the kernel functions used include the Gaussian and polynomial function which are respectively presented in Eq (9) and Eq (10) [37].


$$\gamma \left(\sigma_{k},\sigma \right)=\exp(\frac{{\left|\sigma_{k}-\sigma \right|}^{2}}{d})$$ (9)

$$\gamma (\sigma_{k},\sigma )={\left(\vec{\sigma_{k}}.\vec{\sigma }+1\right)}^{\tau }$$ (10)

Where *d* and *τ* are the Gaussian and polynomial kernel parameters respectively.

### 2.2. Genetic algorithm (GA)

GA is a class of heuristic methods of optimum solutions searching with biological natural selection operational principles [38, 39]. The simplicity of the algorithm coupled with its known and established powerful search mechanisms has ultimately strengthened the application domain of genetic algorithm. The repeating circles of genetic algorithm commence with population initialization in which probable and a set of possible candidates are populated within the solution search space. Each of the possible solution is called an individual within genetic algorithm operation principle description. Iteration stages continuously evolve the initially generated population through the principle of survival of the fitness until weak individuals are replaced with efficient and better ones [40]. Individuals with the associated greater fitness are potentially selected as parents which produce better off springs for the subsequent iteration and the processes continue until optimal individual with optimum solution is established [41]. The genetic operations include selection, crossover and mutation. Each of these operations has associated value of probability which is tuned for controlling the strength and significance of the operation.

## 3.0. Data sample acquisition and computational methodology

This section describes the data sample acquisition and presents stepwise description of the adopted computational methodology and hybridization.

### 3.1. Data sample acquisition

Dysprosium -transition metals based magnetocaloric compounds employed for simulation consist of fifty-four different compounds extracted from literature [10, 13, 42–45]. The predictors to the models include the applied field, ionic radius of the transition metal and that of other incorporated metals or non-metals. Expression presented in Eq (11) shows the dysprosium -transition metal based magnetocaloric compounds that can be fed into the proposed GSVR based models for maximum magnetic entropy change prediction.


$$Dy_{a}T_{b}X_{c}$$ (11)

Where *Dy* is the rare earth dysprosium, T and X are the transition metal and other incorporated metal or non-metal, respectively. The concentration of dysprosium, transition metal and other incorporated metals or non-metal are respectively represented as a,b and c in expression shown in Eq (11). Statistical investigation was conducted on the dysprosium -transition metal based intermetallic compounds and the results of the analysis are presented in Table 1.

**Table 1:** Statistical investigation of the available dysprosium -transition metal based data samples (maximum magnetic entropy change per unit mass, MEC).

Statistical parameters	MEC (J/kg/K)	Applied field (T)	a	T	b	X	c
Mean	8.0056	3.6111	2.3148	56.7037	1.2037	77.2315	1.7593
Maximum	23.0000	7.0000	12.0000	87.0000	4.0000	117.0000	7.0000
Minimum	0.4000	1.0000	1.0000	0.0000	0.0000	30.0000	1.0000
Standard deviation	5.1734	1.6416	2.5015	37.4063	1.0882	22.8094	1.2876
Correlation coefficient	1.0000	0.5057	-0.2121	-0.3035	-0.1678	-0.1805	-0.0173

The presented mean values of all the predictors give insight on the general content of the data samples while the statistical range for each of the predictors and measured maximum magnetic entropy change can be inferred from the maximum and minimum values presented in Table 1. Standard deviation controls the consistency in data samples as the measurements were taken from different dysprosium -transition metals based magnetocaloric compounds under varying experimental conditions. The significance of these statistical parameters becomes manifested during modeling and simulation especially when over/under-fitting set in and the model displays average values of the measured magnetocaloric effect for all its estimates. The presented coefficients of correlation control the level of linearity between the predictors and the maximum magnetic entropy change. Only applied field indicates positive correlation while other predictors show negative values of correlation coefficient and the coefficient values are even low. This means that the degree linearity is weak and this kind of problem needs to be precisely addressed using non-linear model such as the implemented support vector regression algorithm.

### 3.2. Computational methodology

Genetic algorithm is combined with support vector regression for parameter selection and to further strengthen the precision domain of the hybridized model. Computing environment of MATLAB was utilized for hybridization and other sort of simulations involved. The optimized parameters handled by genetic algorithm include the error epsilon, penalty factor and kernel parameter. Computational details are itemized thus:

**Step A:** Data partitioning and randomization. The data sample was separated into training and testing in the ratio of 8:2 while the randomization of data samples precedes data partitioning. Data randomization become necessary purposely to minimize the possible risk of pattern acquisition from set of samples covering limited range of entre data samples and translate to under/over fitting during model validation.

**Step B:** Search space definition and initialization of population. Each gene in genetic algorithm description represents hyper-parameter to be optimized. The search space of each of the parameter is defined and specified as [1000 200,1 0.001,5.9 3.0] for penalty factor, epsilon and kernel parameter, respectively using Gaussian mapping function. The upper search space of penalty factor, epsilon and kernel parameter are 1000, 1 and 5.9, respectively while the lower search space domain are respectively set as 200, 0.001 and 3.0. In a case of polynomial mapping function, the search space was defined as [1000 1,1 0.00001,1 0.00001] for penalty factor, epsilon and kernel parameter, respectively.

**Step C:** Fitness determination. The implemented objective function is the root mean square error (RMSE) of testing samples. The computed fitness procedures are itemized below. (i) Among the pool of potential functions, a function is selected as the kernel function. Other available functions will be selected one of the other until all functions are implemented in a repetitive manner. (ii) The selected kernel function in **step (i)** combines with a chromosome in the initial population generated by genetic algorithm and the training set of samples. The chromosome encodes the parameters to be optimized in a known order (penalty factor, epsilon and kernel parameter). Support vectors are generated at this modeling step. These support vectors are the reproducible models and can be called upon during model validation and future implementation. RMSE between the experimental and estimated values are computed which represent the fitness of the chromosome. RMSE for every chromosome is obtained while the saved support vectors are combined with the testing data samples (iii) The first two **steps (i &ii)** are repeated for other chromosomes of the population. The fitness of each chromosome is ranked while those with lowest value of RMSE are the fit chromosomes. (iv) the entire **steps (i to iii)** are repeated for another selected kernel function and the processes are repeated for all the available functions. The fitness of all the chromosomes in the initially generated population is computed while the best chromosomes are selected for genetic operation and transition to the next generation.

**Step D:** Selection operation: best chromosomes ranked on the basis of fitness function are selected for reproduction and production of offsprings. The selection probability of 0.8 was used. Better offsprings are produced from this modeling stage and constitute the subsequent population.

**Step E:** Crossover operation: This operation allows transference of subsequences and portions from parent chromosome to the new offsprings. 0.9 crossover probability was for ensuring possible replacement of less fit chromosomes with better ones in the subsequent population replacement.

**Step F:** Mutation operation. This operation changes and alters string random positioning and the operation was set at 0.005 probability value.

**Fig 1:**
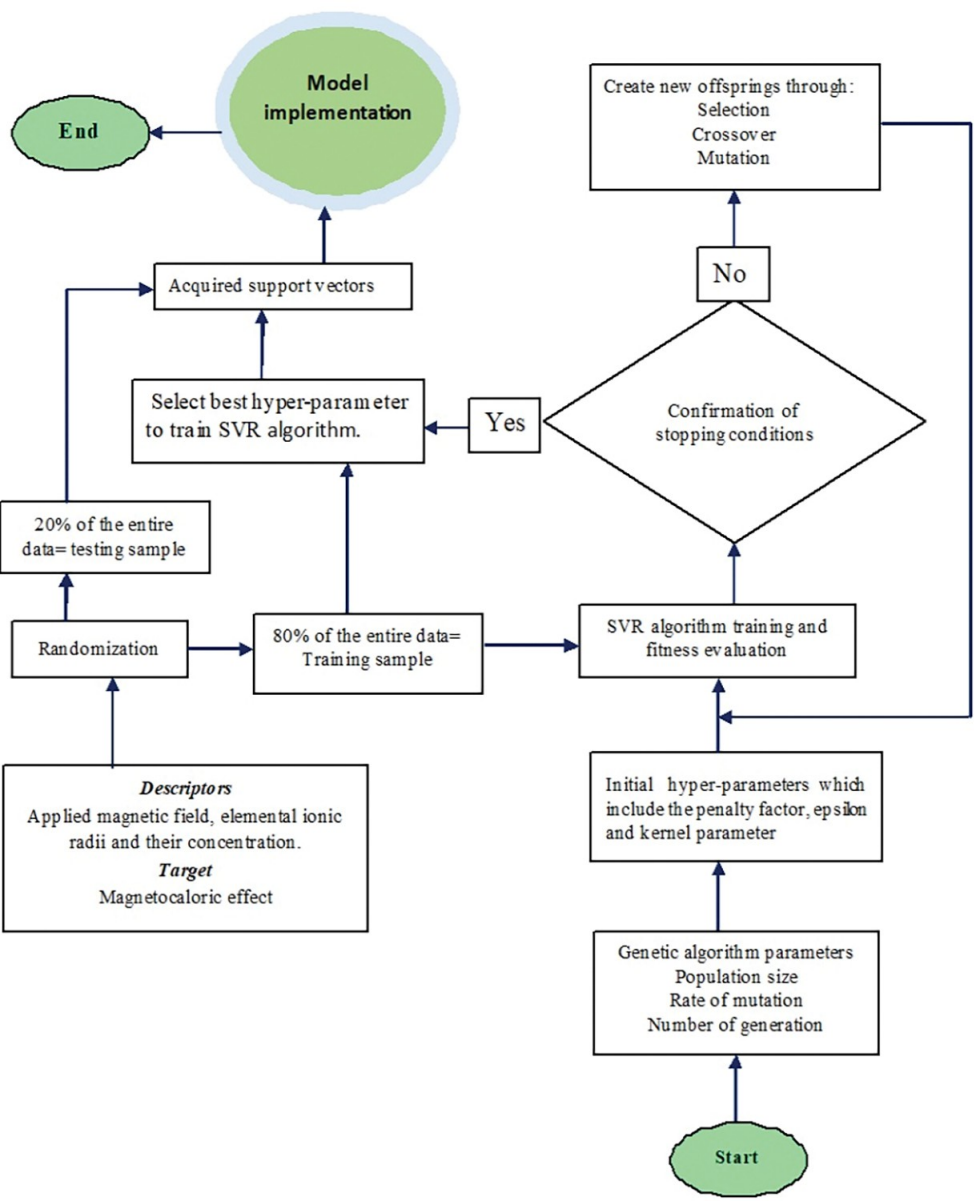
Computational procedures for GSVR based models.

**Step G:** Stopping conditions. These conditions include attainment of (i) zero value of RMSE (ii) maximum number of iteration and (iii) fifty consecutive iterations with the same value of testing RMSE. **Fig 1** shows the computational chat of the hybrid algorithm.

## 4.0. Results and discussion

This section presents the convergence of genetic algorithm at different iterations for two different mapping functions. The predictions of the two models also presented with performance assessment parameters for each of the model. Results of the investigation of the effect of applied magnetic field on dysprosium -transition metals based magnetocaloric compounds are also discussed.

### 4.1. Optimization of model parameters using genetic algorithm

**Fig 2:**
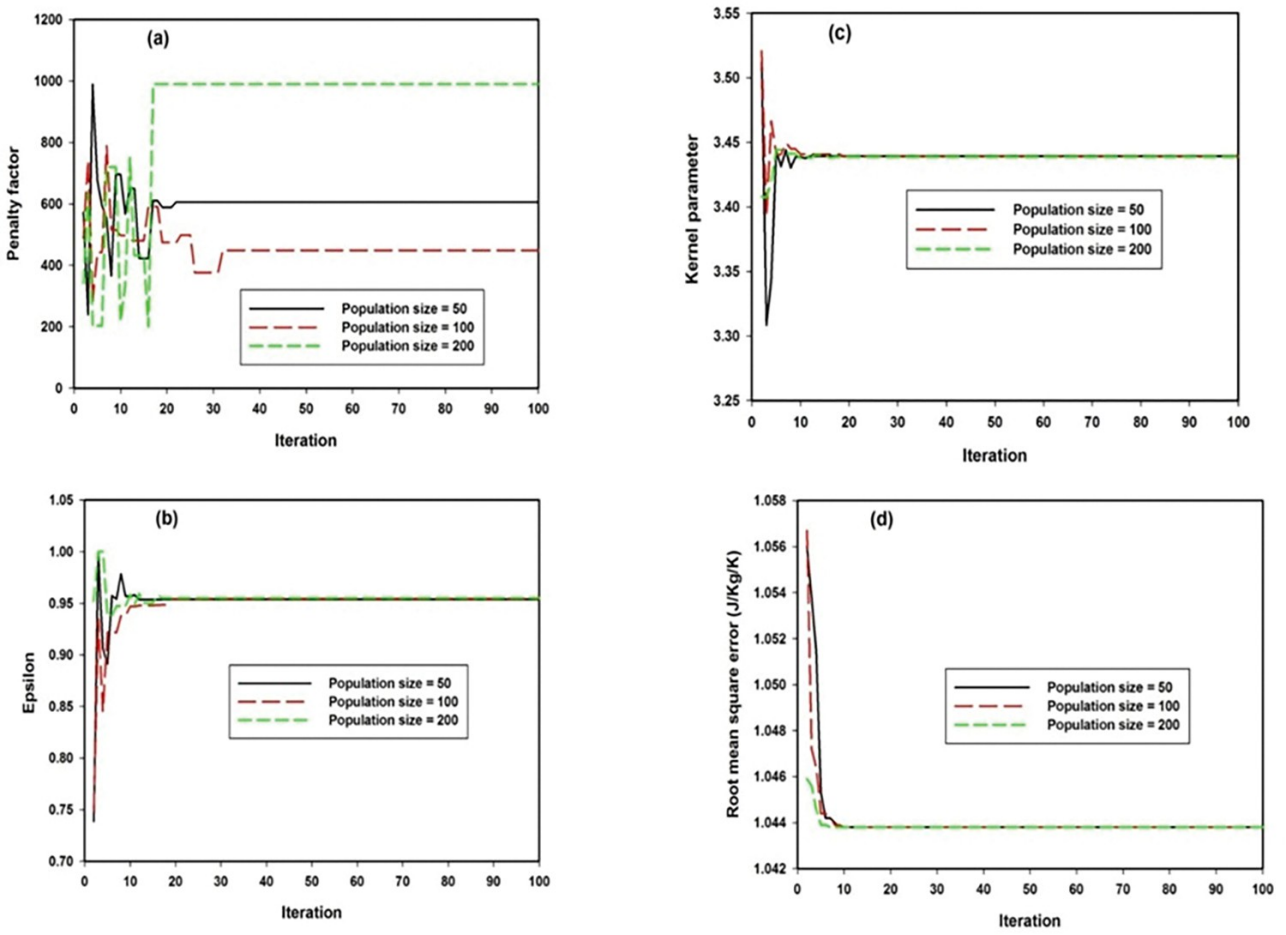
Convergence parameters for GSVR-G model at three different population sizes (a) penalty factor (b) epsilon error threshold (c) kernel parameter for Gaussian mapping function (d) RMSE.

The convergences of penalty factor, epsilon, Gaussian mapping function and model fitness function are shown in **Fig 2** for three different population size. Penalty factor convergence shown in **Fig 2A** indicates random convergence for iteration below twenty for population size of fifty and two-hundred while random convergence still persists for population size of one hundred up to iteration size close to forty. During this stage, the optimization algorithm explores the solution search space and further exploits space for optimum solution convergence.

The model convergence continues until the iteration size attains one hundred. Similar convergence behavior and pattern are demonstrated by epsilon error and Gaussian kernel parameter presented in **Fig 2B** and **2C**, only that all the population size converged to the same optimization point for iteration above ten. The convergence of model fitness as contained in RMSE is presented in **Fig 2D** for different iteration number. Similar pattern of convergence is demonstrated by all population sizes after ten iteration number which signifies robustness of the developed GSVR-G model.

**Fig 3:**
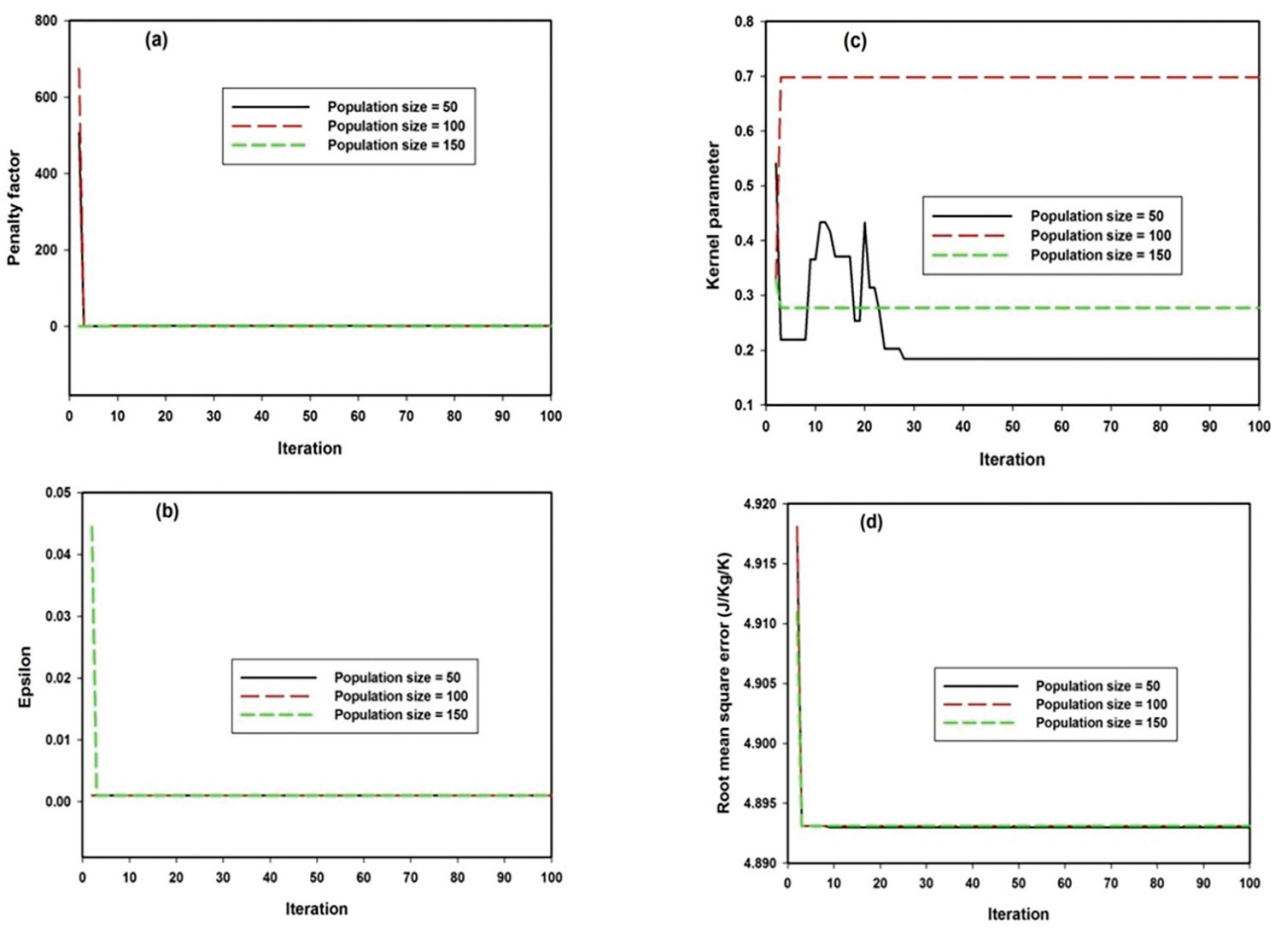
Convergence parameters for GSVR-P model at three different population sizes (a) penalty factor (b) epsilon error threshold (c) kernel parameter for polynomial mapping function (d) RMSE.

Convergence of model’s parameters in the case of GSVR-P model is presented in **Fig 3** for population sizes of fifty, one hundred and one-hundred and fifty. The convergence presented in **Fig 3A** for penalty factor shows similar convergence for population sizes of 50,100 and 150 especially when iteration is above five. Similar convergence pattern has been demonstrated by epsilon convergence presented in **Fig 3B**. Kernel parameter for polynomial mapping function is presented in **Fig 3C** while random convergence was observed for population size of 50 when iteration is below 30. Randomness of convergence pattern reduces with increase in population size to 100 and 150 and uniform convergence was attained after ten number of iteration. Error convergence is presented in **Fig 3D** for different iteration sizes and robust convergence is attained for all the population sizes when number of iteration is below ten. Table 2 presents optimum values for each of the model parameters associated with the developed hybrid models.

**Table 2:** Model parameters and their associated optimum solutions.

Model	Epsilon	Population size	Mapping function	Penalty factor	Kernel parameter
GSVR-G	0.9537	50	Gaussian	606.2589	3.4393
GSVR-P	0.001	50	Polynomial	1.2713	0.184

### 4.2. Performance assessment parameters associated with the developed hybrid models

The prediction and generalization performance of GSVR-G and GSVR-P models evaluated using mean absolute error (MAE), correlation coefficient (CC) and root mean square error (RMSE) for training and testing dysprosium-transition metal based magnetocaloric compounds. The developed GSVR-G shows 0.91 J/kg/K, 99.05% and 0.92 J/kg/K values of MAE, CC and RMSE, respectively using training magnetocaloric samples while its counterpart GSVR-P model demonstrates performance value of 2.89 J/kg/K for MAE, 68.58% for CC and 4.05 J/kg/K for RMSE. It shows that GSVR-G performs better then GSVR-P using training magnetocaloric samples. For testing dysprosium -transition metal based magnetocaloric compounds, GSVR-G shows 0.87 J/kg/K, 93.35% and 1.04 J/kg/K for MAE, CC and RMSE, respectively while the respective obtained assessment values for GSVR-P model are 4.10 J/kg/K, 13.78% and 4.89 J/kg/K. Table 3 presents the values for all performance assessment parameters for all set of samples.

**Table 3:** Performance assessment parameters and their comparison for GSVR-G and GSVR-P models.

Hybrid model	Training			Testing		
	CC	MAE	RMSE	CC	MAE	RMSE
GSVR-G	0.9905	0.9104	0.9217	0.9335	0.8697	1.0438
GSVR-P	0.6858	2.8896	4.0515	0.1378	4.1061	4.893
% improvement of GSVR-G over GSVR-P	30.7622	68.4939	77.2504	85.2384	78.8193	78.6675

**Fig 4:**
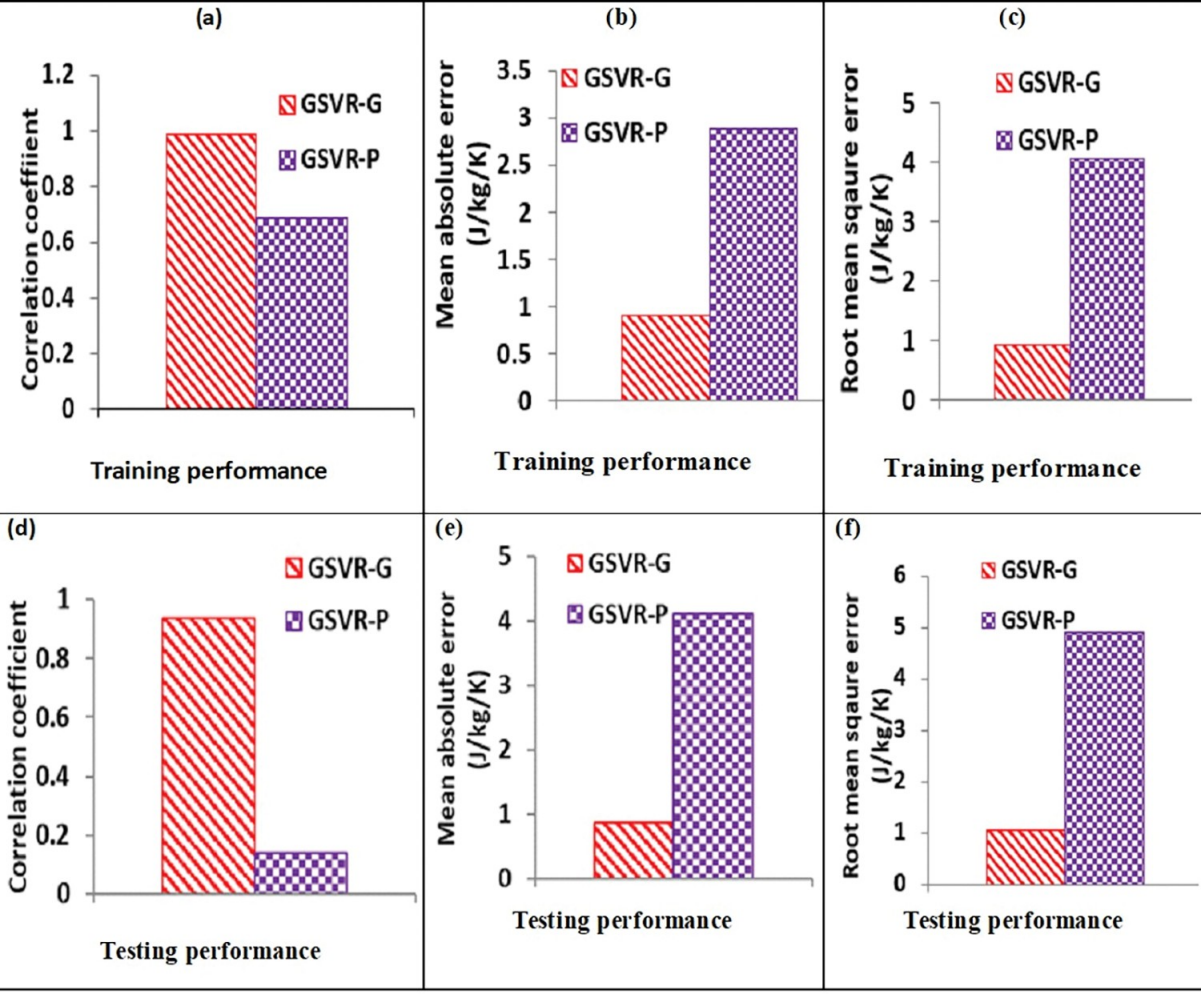
Performance assessment parameters for GSVR-G and GSVR-P models using (a) CC metric for training samples (b) MAE metric for training samples (c) RMSE metric for training samples (d) CC metric for testing sample (e) MAE metric for testing sample (f) RMSE metric for testing sample.

Performance improvement demonstrated by GSVR-G model over GSVR-P model is also presented in Table 3. The developed GSVR-G model outperforms GSVR-P model with improvement of 30.76%, 68.49% and 77.25% for training dysprosium -transition metal based magnetocaloric compounds as respectively presented in **Fig 4A** using CC metric, **Fig 4B** using MAE metric and **Fig 4C** using RMSE metric. Assessment of training dysprosium -transition metal based magnetocaloric compounds shown **Fig 4D–4F** indicate superior performance of GSVR-G over GSVR-P with improvement of 85.23% for CC metric, 78.82% for MAE metric and 78.67% for RMSE metric, respectively.

### 4.3. Predictions of GSVR-G and GSVR-P model and the corresponding absolute error for dysprosium transition metal based intermetallic magnetocaloric compounds

Table 4 presents the predictions of the developed GSVR-G and GSVR-P models for magnetocaloric effect of dysprosium transition metal based intermetallic compounds with their associated absolute errors. The estimates of GSVR-G model are closer to the measured values with slight deviations as compared with the predictions of GSVR-P model. The computed mean absolute percentage deviation (MAPD) for developed GSVR-G model and GSVR-P are 0.9 J/kg/K and 3.1 J/kg/K, respectively. This indicates the precision of GSVR-G model over GSVR-P model due to the employed Gaussian mapping function for data sample transformation. Superior performance of the Gaussian based model can be attributed to the mathematical formulation of Gaussian function as compared with polynomial function.

**Table 4:** Estimated magnetocaloric effect (maximum magnetic entropy change per unit mass, MEC) for different dysprosium transition metal based intermetallic compounds.

Compound	Field(T)	MEC (J/kg/K)	GSVR-G (J/kg/K)	Absolute error	GSVR-P (J/kg/K)	Absolute error
DyCuAl	2	10.9 [42]	11.9	1.0	3.9	7.0
DyCuAl	5	20.4 [42]	19.4	1.0	9.5	10.9
Dy_2_Cu_2_Cd	2	7.2 [43]	8.2	1.0	1.8	5.4
Dy_2_Cu_2_Cd	5	13.8 [43]	12.8	1.0	7.4	6.4
Dy_2_Cu_2_In	2	7.2 [43]	8.2	1.0	2.5	4.7
Dy_2_Cu_2_In	5	13.3 [43]	12.3	1.0	8.1	5.2
Dy_2_Co_2_Ga	2	3.2 [43]	4.2	1.0	3.9	0.7
Dy_2_Co_2_Ga	5	6.2 [43]	7.9	1.7	9.4	3.2
DyFeAl	5	6.4 [10]	6.2	0.2	8.4	2.0
DyCoNi	5	9.9 [13]	8.9	1.0	9.9	0.0
DyNiSn	1	0.4 [45]	1.4	1.0	1.6	1.2
DyNiSn	2	1.6 [45]	2.2	0.6	3.4	1.8
DyNiSn	3	3.5 [45]	4.5	1.0	3.5	0.0
DyNiSn	5	8.0 [45]	7.0	1.0	9.0	1.0
DyNiSb	2	2.6 [45]	3.6	1.0	3.1	0.5
DyNiSb	3	5.2 [45]	4.2	1.0	5.0	0.2
DyNiSi	2	12.1 [45]	11.1	1.0	4.8	7.3
DyNiSi_3_	5	5.3 [45]	6.3	1.0	9.6	4.3
Dy_2_Ni_3_Si_5_	5	7.9 [45]	7.3	0.6	9.6	1.8
DyNiSi_2_	5	4.1 [45]	5.1	1.0	10.8	6.7
DyNi_4_Si	1	3.6 [45]	4.6	1.0	3.9	0.3
DyNi_4_Si	5	11.3 [45]	10.3	1.0	11.3	0.0
DyCo_3_B_2_	1	3.5 [45]	3.7	0.2	5.3	1.8
DyCo_3_B_2_	2	6.6 [45]	5.9	0.7	5.4	1.2
DyCo_2_B_2_	2	5.4 [45]	5.9	0.5	5.4	0.0
DyCo_2_B_2_	5	12.1 [45]	11.1	1.0	10.9	1.2
Dy_2_CoGa_3_	5	10.8 [45]	9.8	1.0	8.7	2.1
Dy_5_Pd_2_	7	8.3 [45]	8.4	0.1	15.9	7.6
Dy_3_Co	5	13.9 [45]	12.9	1.0	13.9	0.0
DyNi	5	18.0 [45]	19.0	1.0	14.1	3.9
DySb	5	15.8 [45]	14.8	1.0	13.8	2.0
DySi	5	6.8 [45]	7.8	1.0	15.5	8.7
DyGa	2	2.3 [45]	3.3	1.0	8.9	6.6
DyGa	5	5.8 [45]	6.6	0.8	14.5	8.7
DyCu_2_	5	10.1 [45]	11.1	1.0	13.6	3.5
DyAl_2_	1	5.4 [45]	6.4	1.0	7.1	1.7
DyAl_2_	2	9.9 [45]	8.9	1.0	8.9	1.0
DyNi_2_	2	11.9 [45]	12.9	1.0	8.2	3.7
DyNi_2_	5	23.0 [45]	22.0	1.0	13.8	9.2
DyB_2_	2	7.4 [45]	8.4	1.0	10.1	2.7
DyB_2_	5	17.0 [45]	16.0	1.0	15.7	1.3
Dy1_2_Co_7_	2	4.9 [45]	5.9	1.0	4.4	0.5
Dy_12_Co_7_	5	10.0 [45]	9.0	1.0	10.0	0.0
Dy_2_Ni_2_In	2	1.1 [45]	2.1	1.0	2.8	1.7
Dy_2_Ni_2_In	5	6.4 [45]	8.3	1.9	10.2	3.8
Dy_2_Cu_2_Cd	2	7.2 [45]	8.2	1.0	1.8	5.4
Dy_2_Cu_2_Cd	5	13.8 [45]	12.8	1.0	7.4	6.4
Dy_2_Cr_2_C_3_	5	13.5 [45]	12.5	1.0	10.3	3.2
Dy_6_FeBi_2_	2	1.7 [45]	2.7	1.0	1.0	0.7
Dy_6_FeBi_2_	5	3.8 [45]	4.1	0.3	6.6	2.8
Dy_6_MnBi_2_	2	0.6 [45]	1.6	1.0	0.6	0.0
Dy_6_MnBi_2_	5	1.3 [45]	2.3	1.0	6.2	4.9
Dy_6_FeSb_2_	2	3.0 [45]	4.0	1.0	2.2	0.8
Dy_6_FeSb_2_	5	6.9 [45]	5.9	1.0	7.8	0.9
**Mean absolute percentage deviation (MAPD)**				**0.9**		**3.1**

### 4.4. Investigating the influence of applied magnetic field on magnetocaloric effect of DyCuAl dysprosium transition metal based intermetallic compound using GSVR-G model

**Fig 5:**
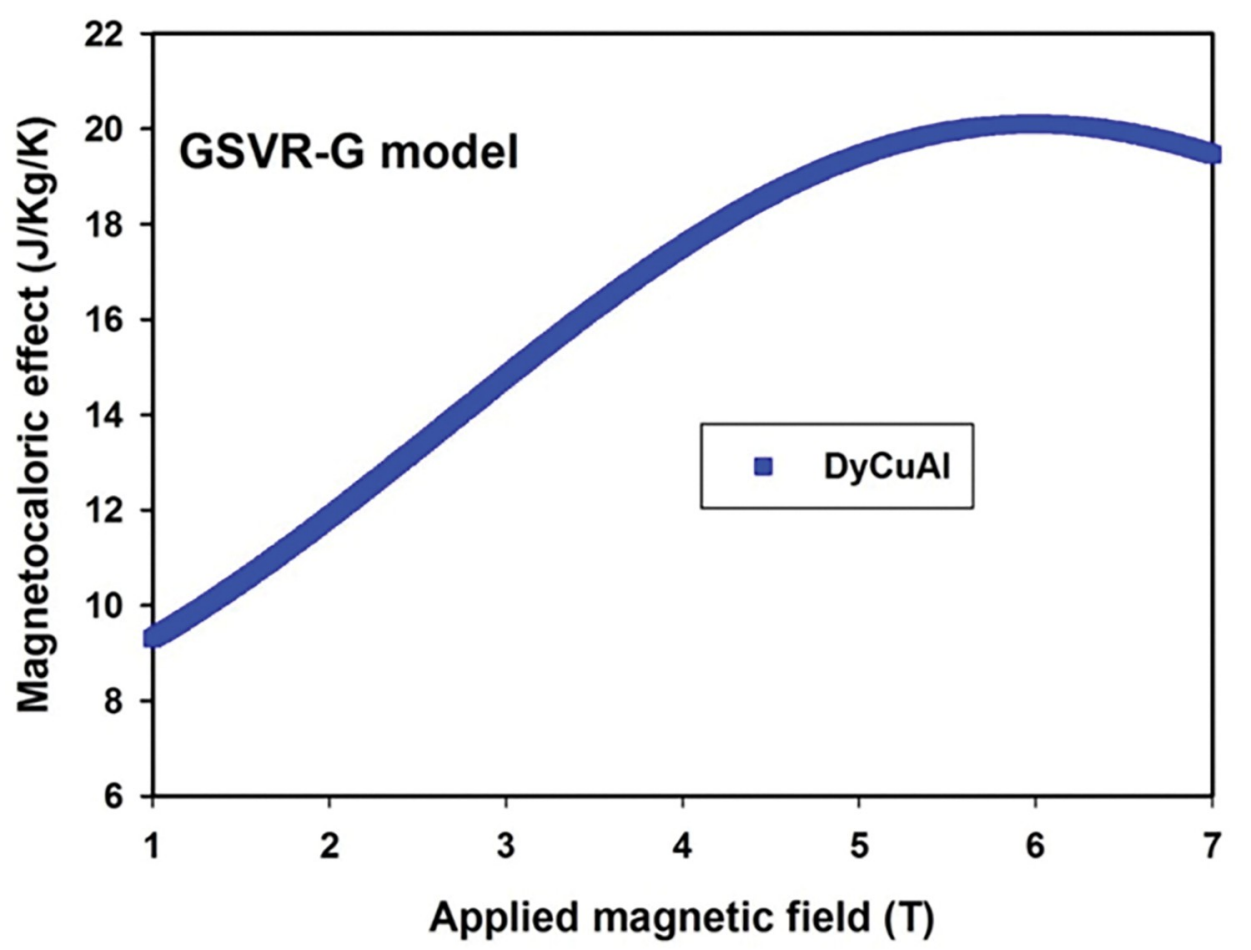
Response of magnetocaloric effect of DyCuAl compound at different applied field using GSVR-G model.

The developed GSVR-G model was employed to investigate how applied magnetic field affect magnetocaloric effect of DyCuAl compound and the response of the developed GSVR-G model is depicted by **Fig 5**. During this implementation, GSVR-G model was supplied with only the predictors while the saved support vectors during pattern acquisition stage were employed for the predictions. The magnetocaloric effect of DyCuAl compound was found to increase with increase in applied magnetic field up to the field of 6T after which increase in the strength of the field lowers the magnetocaloric effect demonstrated by the compound.

## 5. Conclusion

Magnetocaloric effect of dysprosium transition metal based intermetallic magnetocaloric compounds are modeled through genetic algorithm-based support vector regression (GSVR) intelligent computational method. The developed hybrid model employed both Gaussian (G) and polynomial (P) mapping function for data sample transformation. The descriptors to the models include the ionic radii of the constituting elements, their concentrations, and the applied magnetic field. Using performance assessment parameters such as the correlation coefficient CC, mean absolute error (MAE) and root mean square error (RMSE), the developed GSVR-G model outperforms GSVR-P model using training and testing dysprosium transition metals based magnetocaloric compounds. The developed GSVR-G model outperforms GSVR-P model with improvement of 30.76%, 68 .49% and 77.25% for training dysprosium -transition metal based magnetocaloric compounds using CC, MAE and RMSE metrics, respectively while performance improvement of 85.23% for CC metric, 78.82% for MAE metric and 78.67% for RMSE metric were obtained for testing set of dysprosium transition metals based intermetallic compounds. The influence of applied field on magnetocaloric effect of DyCuAl compound was investigated using developed model through support vectors implementation. Closeness of the predictions of the developed model, circumvention of experimental difficulties involved in magnetocaloric effect quantification, quick predictions with reduced cost and utilization of simple descriptors are among the unique features of the developed model that would potentially facilitate exploration of these compounds for cooling applications.

## Supporting information

S1 Data (XLSX)
